# Roles of exosome-mediated macrophage polarization in the hepatocellular carcinoma progression

**DOI:** 10.3389/fimmu.2026.1907211

**Published:** 2026-08-17

**Authors:** Anna Chen, Minmin Zhu

**Affiliations:** Department of Infectious Disease, The Fourth Affiliated Hospital of School of Medicine, and International School of Medicine, International Institutes of Medicine, Zhejiang University, Yiwu, China

**Keywords:** exosomes, hepatocellular carcinoma, immunotherapy, macrophage polarization, tumor microenvironment, tumor-associated macrophages

## Abstract

Hepatocellular carcinoma (HCC) remains a leading cause of cancer-related mortality worldwide. The tumor microenvironment (TME) is a highly immunosuppressive niche shaped by aberrant angiogenesis, chronic inflammation, and extracellular matrix remodeling, which facilitates immune evasion and promotes HCC proliferation, invasion, and metastasis. Tumor-derived exosomes act as critical mediators of intercellular communication, delivering bioactive cargo such as microRNAs (miRNAs) and long non-coding RNAs (lncRNAs) to macrophages, thereby modulating their polarization and functional state. Specifically, exosomal signals induce M2 macrophage polarization, promoting tumor progression, immune suppression, and metastatic dissemination, whereas M1 macrophage polarization enhances antitumor immunity via activation of cytotoxic T cells and pro-inflammatory signaling. Key regulatory pathways include STAT3 activation and NF-κB signaling, which govern exosome-mediated macrophage reprogramming and cytokine release, further modulating the TME. This review summarizes the current insights into exosome-driven macrophage polarization in HCC, providing a potential immunotherapeutic strategy for restoring antitumor immunity and improving HCC patient prognosis.

## 1 Introduction

Hepatocellular carcinoma (HCC) is one of the most common causes of cancer-related mortality (1). Understanding how HCC cells communicate with immune and stromal components of the tumor microenvironment (TME), particularly through exosome-mediated regulation of macrophage polarization, is essential for developing more effective therapeutic strategies for HCC (2). The HCC TME is an immunosuppressive niche shaped by aberrant angiogenesis, chronic inflammation, and remodeled extracellular matrix components, thereby facilitating immune evasion and promoting HCC proliferation, invasion, and metastasis (3, 4). The formation and evolution of the TME therefore play a critical role in HCC initiation, progression, and therapeutic response. Exosomes, as important mediators of intercellular communication, interact with macrophages and promote their polarization toward an M2-like phenotype, thereby driving the HCC TME into an immunosuppressive, tumor-promoting state (5–7). Moreover, recent advances in single-cell profiling have revealed substantial heterogeneity and dynamic polarization states among tumor-associated macrophages in HCC, underscoring the need to systematically examine how exosome-mediated signals reshape macrophage phenotypes within the TME. This review summarizes current research progress on exosome-induced macrophage polarization within the HCC TME, with the aim of providing a theoretical basis for the clinical development of more effective exosome-based biomarkers and therapeutic strategies.

## 2 Overview of the tumor microenvironment and exosomes

The tumor microenvironment (TME) refers to the cellular and non-cellular milieu required for the survival, growth, proliferation, and metastatic dissemination of malignant cells (8, 9). In recent years, the TME has been recognized as a critical determinant of multiple stages of tumor biology, including tumor growth, metastasis, therapeutic resistance, cancer diagnosis, and targeted therapy (10). Consequently, the TME has become a major focus in basic and translational cancer research. The TME comprises tumor cells, infiltrating immune cells, stromal components, extracellular matrix, and soluble mediators. Infiltrating immune cells include macrophages, dendritic cells, natural killer (NK) cells, T cells, and lymphocytes. In contrast, stromal and extracellular components include cancer-associated fibroblasts (CAF), endothelial cells, lipid components, and extracellular matrix. Soluble mediators, including growth factors, inflammatory cytokines, and other signaling molecules, further shape cell–cell communication within the TME (9, 11). Among these components, tumor-associated macrophages (TAMs) constitute one of the predominant non-malignant cells within the TME and are major regulators of tumor progression, immune suppression, angiogenesis, and metastatic dissemination (12–14). Because TAM polarization is strongly influenced by signals derived from tumor and stromal cells, intercellular communication within the TME is essential for shaping macrophage phenotype and function. Exosomes are important mediators of this communication, as they possess intact membrane structures, exhibit high stability in biological fluids, and transport bioactive molecules from tumor cells, immune cells, fibroblasts, and other cell types to recipient cells, thereby regulating the biological behavior of target cells (15–17).

Exosomes are extracellular vesicles with a lipid bilayer membrane structure and a diameter of approximately 40–160 nm that contain diverse bioactive cargo derived from parental cells, including RNA molecules such as microRNA (miRNA), messenger RNA (mRNA), and long non-coding RNA (lncRNA), as well as cytokines, growth factors, proteins, lipids, and DNA (18–20). In HCC, exosomes have been detected in clinically relevant biological fluids such as blood, ascites, and bile, supporting their potential application as minimally invasive biomarkers (21). Exosomes can be released by both normal and tumor cells and can shuttle between different cell types to mediate local and long-distance intercellular communication (22). Accumulating evidence indicates that exosomes are key mediators of macrophage phenotypic remodeling (23, 24). In the TME, exosomes participate in multiple biological processes that promote malignant progression. For example, they can enhance tumor angiogenesis, regulate inflammatory signaling, and mediate extracellular matrix remodeling, thereby contributing to local tumor proliferation, invasion, and distant metastasis (25). These properties suggest that exosomes are not only important regulators of tumor–stromal and tumor–immune interactions, but also potential biomarkers and therapeutic targets in cancer progression.

## 3 Exosome-mediated regulation of the HCC TME through macrophage polarization

### 3.1 Macrophages in the TME

**Figure 1:**
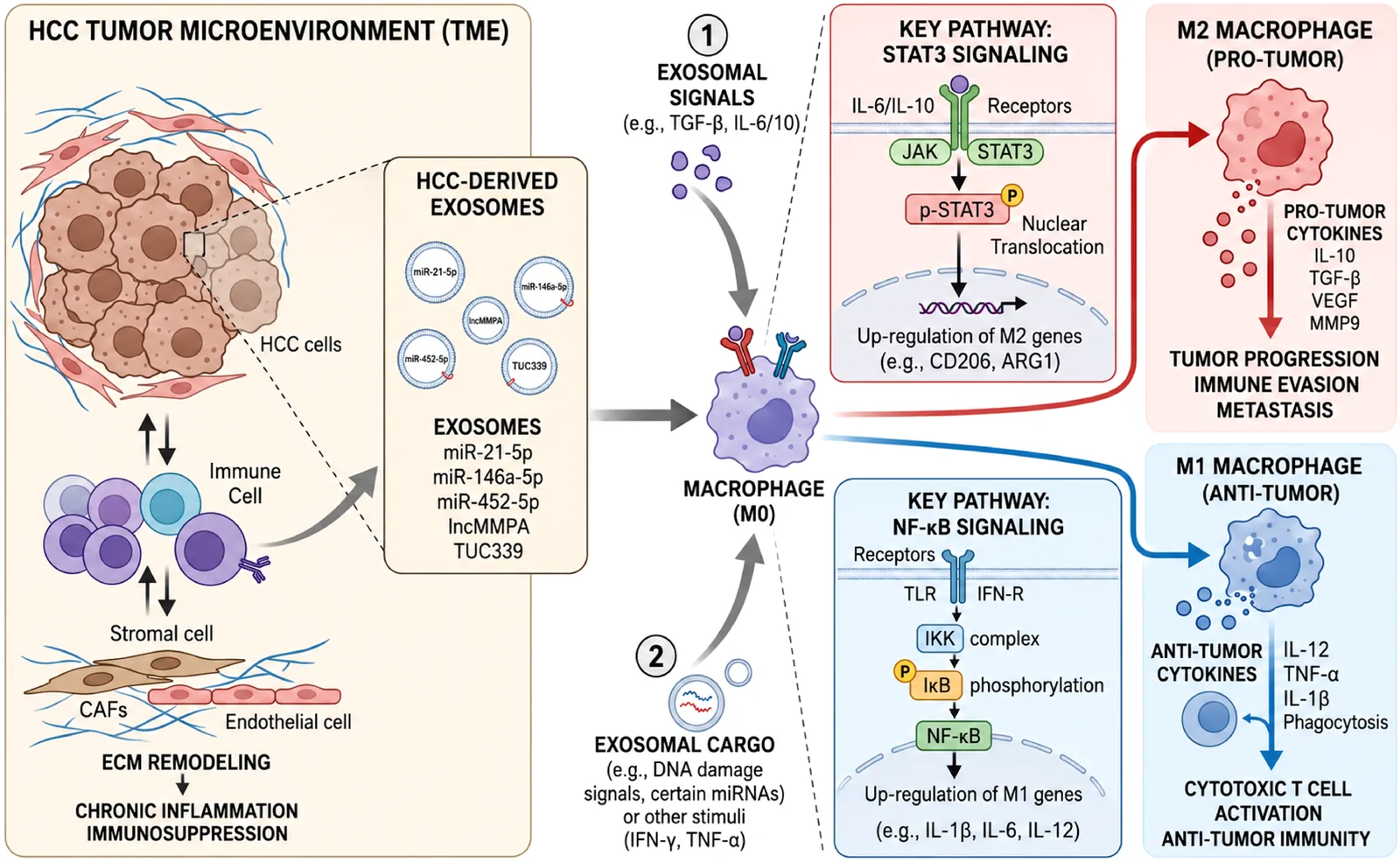
Exosome-mediated regulation of the HCC progression. HCC-derived exosomes act as important mediators of intercellular communication within the hepatocellular carcinoma (HCC) tumor microenvironment (TME). Exosomes released by HCC cells carry bioactive cargo, including miR-21-5p, miR-146a-5p, miR-452-5p, LncMMPA, and TUC339, which can be transferred to macrophages and regulate their polarization. In the immunosuppressive HCC TME, exosomal signals such as TGF-β, IL-6, and IL-10 activate the JAK/STAT3 pathway, leading to STAT3 phosphorylation, nuclear translocation, and upregulation of M2-associated genes such as CD206 and ARG1. M2-like macrophages subsequently secrete pro-tumor cytokines and factors, including IL-10, TGF-β, VEGF, and MMP9, thereby promoting tumor progression, immune evasion, angiogenesis, and metastasis. In contrast, exosomal cargo or inflammatory stimuli such as IFN-γ and TNF-α may activate NF-κB signaling through TLR or IFN-R-mediated pathways, inducing M1-associated gene expression and enhancing anti-tumor immune responses. M1 macrophages produce anti-tumor cytokines, including IL-12, TNF-α, and IL-1β, promote phagocytosis, and facilitate cytotoxic T-cell activation.

Macrophages are among the most prominent infiltrating immune cells within the TME and exhibit remarkable plasticity in response to distinct environmental stimuli (26, 27). This plasticity enables macrophages to adopt functionally divergent phenotypes, including classically activated M1 macrophages and alternatively activated M2 macrophages (28). M1 and M2 macrophages possess distinct gene expression profiles. Functionally, M1 macrophages are generally considered immunostimulatory, being activated by IFN-γ, TNF-α, and secret pro-inflammatory cytokines, which promote immune and anti-infective activities and support both inflammatory and antitumor functions (29, 30). In contrast, M2 macrophages secrete factors such as IL-4 and IL-13, thereby promoting angiogenesis and fibrogenesis, suppressing cytotoxic T-cell activity, and ultimately facilitating tumor progression through immunosuppressive effects (31, 32). Thus, in cancer, macrophages function as a double-edged sword. The dynamic balance of M1/M2 polarization significantly influences the remodeling of the local tissue microenvironment. Local tumor recurrence and distant metastasis are not solely determined by malignant cell behavior; macrophages are key drivers of these processes (33). Evidence suggests that recruitment of M1 macrophages into the TME correlates with reduced tumor invasiveness, whereas accumulation of M2 macrophages is associated with enhanced tumor progression and poor prognosis (34). Higher M1/M2 ratios have also been linked to longer overall survival in cancer patients (35, 36). Once TAMs adopt an M2 phenotype within the TME, they can promote tumor progression via immune suppression, tissue remodeling, and angiogenesis (37) (**Figure 1**).

### 3.2 M1 Macrophage in HCC progression

Previous studies have reported that promoting TAM polarization toward an M1-like phenotype can suppress HCC progression and improve prognosis in both *in vivo* models and clinical studies (38, 39). M1 macrophages exert antitumor immune activity within the TME by enhancing macrophage phagocytosis and cytotoxic T lymphocyte activity (40, 41). Nuclear factor-κB (NF-κB) is a central transcriptional regulator of inflammatory macrophage activation and is closely associated with the expression of M1-related cytokines, including TNF-α, IL-1β, and IL-6. Activation of NF-κB signaling can therefore enhance antitumor inflammatory responses and promote M1-like macrophage polarization in the HCC TME. *In vitro*, NAD-dependent deacetylase in HCC cells can regulate M1 polarization through the NF-κB pathway, thereby inhibiting HCC metastasis and promoting tumor-cell apoptosis (42). The overexpression of retinoic acid-inducible gene I (RIG-I) promotes M1 macrophage polarization by activating NF-κB signaling. *In vivo*, RIG-I-induced M1 macrophages enhanced HCC cell apoptosis and suppressed tumor growth (43).

HCC-derived exosomes can also influence M1 macrophage polarization through specific cargo molecules. Researchers discovered that HCC-derived exosomes could promote macrophage polarization toward the M1 phenotype through paracrine signaling and thereby exert antitumor immune effects (44). Specifically, HCC-derived exosomal miR-144 and miR-451a were highly expressed and contributed to M1 macrophage polarization. Mechanistically, miR-144 and miR-451a enhanced antitumor activity by negatively regulating hepatocyte growth factor (HGF) and macrophage migration inhibitory factor (MIF), respectively, while suppressing cytokines associated with M2 polarization (44). *In vivo*, miR-142-3p was highly expressed in HBV^+^ HCC cells and M1 macrophages, Mechanistically, miR-142-3p promoted ferroptosis in HBV^+^ M1 macrophages by targeting SLC3A2, thereby accelerating HCC progression (45). Conversely, knockdown of exosomal miR-142-3p reversed ferroptosis in M1 macrophages and inhibited the proliferation, migration, and invasion of HCC cells.

In addition, M1 macrophage abundance has been reported to positively correlate with responses to anti-PD-1 therapy (46, 47). In HCC, high levels of DNA damage can activate the cGAS-STING pathway in M1 macrophages within the TME (48). Activated M1 macrophages can recruit T lymphocytes into tumors, thereby increasing the sensitivity of HCC to anti-PD-1 therapy (49, 50). TACE-resistant HCC can also reshape the tumor immune microenvironment by reducing the secretion of exosomal miR-32-5p. Importantly, miR-32-5p promotes macrophage polarization toward the tumor-suppressive M1 phenotype by increasing the production of M1-associated pro-inflammatory cytokines (51). Notably, M1 macrophages may activate the NF-κB signaling pathway to regulate the expression of cell cycle-related proteins, thereby promoting HCC cell proliferation and reducing the efficacy of postoperative TACE in patients with HCC (52).

### 3.3 M2 macrophage in HCC progression

The principal mechanism by which exosomes regulate macrophage polarization in the HCC TME involves the delivery of tumor-derived exosomal cargo, including miRNAs and lncRNAs, to recipient macrophages. These bioactive molecules can reprogram macrophages toward an M2-like phenotype and thereby promote tumor progression (53–55). Exosomal miRNAs are key regulators of macrophage polarization, phagocytic activity, inflammatory activation, and metabolic remodeling (56, 57). Studies have shown that HCC-derived exosomes are enriched in miR-146a-5p. Suppression of miR-146a-5p increased TNF-α levels and markedly reduced the M2 marker CD206 (58, 59). Mechanistically, miR-146a-5p regulates p-STAT3 and C/EBPβ signaling, thereby reversing M2 macrophage polarization (60, 61). These findings confirm that exosomal miR-146a-5p derived from HCC cells mediates M2 macrophage polarization. In addition, SALL4 has been shown to bind the promoter of miR-146a-5p and regulate its expression, thereby participating in M2 macrophage polarization and improving prognosis in HCC mouse models (62, 63). miR-21-5p is highly expressed in hepatoma cells associated with poor prognosis (5). Overexpressed miR-21-5p negatively regulates RhoB, thereby modulating the MAPK signaling pathway in macrophages. This pathway is involved in the production of the protumor factor IL-10 and promotes M2-like macrophage polarization. Co-culture of macrophages with exosomal miR-1260b derived from hypoxic HCC cells significantly increased the expression of the M2 macrophage marker CD206 after uptake by M0 macrophages (64). These results indicate that hypoxic HCC cell-derived exosomal miR-1260b can induce M2 macrophage polarization, and that inhibition of miR-1260b may potentially reverse macrophage polarization from the M2 phenotype toward the M1 phenotype. Other studies have demonstrated that miR-149-5p negatively regulates M2 macrophage-mediated promotion of HCC invasion and metastasis by targeting MMP9. Specifically, miR-149-5p can bind to the 3′ untranslated region of MMP9 mRNA, thereby reversing the protumor effects of M2 macrophages on HCC cells (65). HCC-derived exosomal miR-452-5p positively regulates CD206, IL-10, and arginase-1 expression, thereby inducing macrophage polarization toward the M2 phenotype and promoting HCC progression (7). Most of these miRNAs have been identified and validated through bioinformatics screening and enrichment analyses.

Beyond miRNAs, exosomal lncRNAs provide another layer of post-transcriptional regulation that links macrophage polarization, metabolic remodeling, and immune suppression in HCC (66, 67). TAM-derived exosomal lncRNAs may enhance aerobic glycolysis in HCC cells (6, 68). For example, the myeloid-specific lncRNA LncMMPA stabilizes ALDH1A3, thereby enhancing aerobic glycolysis and promoting HCC cell proliferation (6). Another study showed that lncRNAs are enriched in HCC-derived exosomes. The lncRNA TUC339 functions as a novel signaling mediator that suppresses macrophage phagocytosis by negatively regulating pro-inflammatory factors and Fcγ receptors, while increasing the expression of chemokines and CD206 (69). Notably, modulation of TUC339 levels can regulate bidirectional conversion between M1 and M2 macrophages: knockdown of TUC339 promotes the transition from M2 to M1 macrophages, whereas overexpression of TUC339 drives the conversion from M1 to M2 macrophages (70, 71). Together, HCC-derived exosomal miRNAs and lncRNAs promote M2-like macrophage polarization, suppress antitumor immunity, and facilitate HCC proliferation, invasion, and metastasis (5, 7, 62).

**Figure 2:**
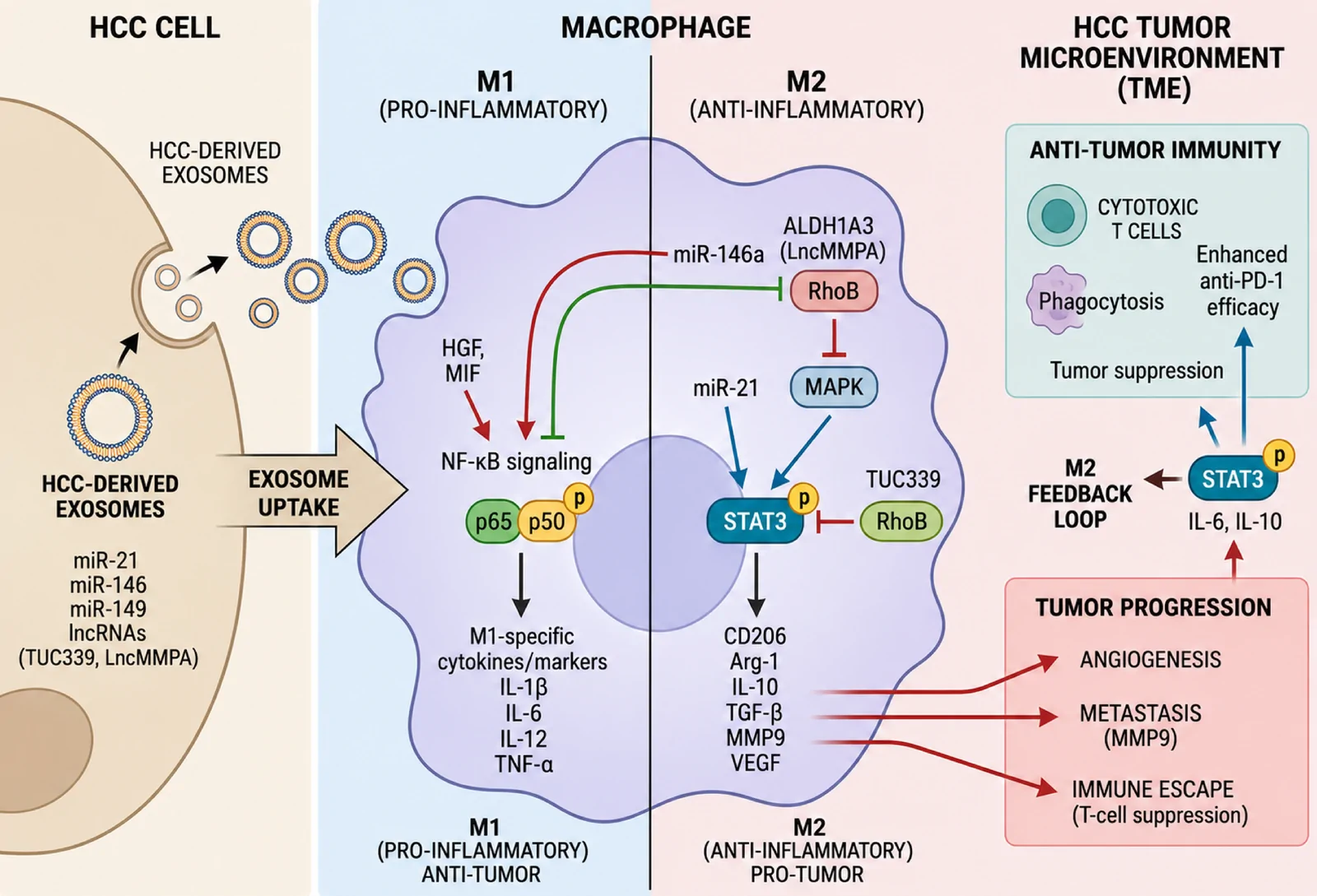
Exosome-mediated macrophage polarization modulates hepatocellular carcinoma tumor microenvironment. HCC-derived exosomes transfer bioactive miRNAs and lncRNAs to macrophages and regulate their polarization. Activation of NF-κB signaling promotes the M1 phenotype and induces pro-inflammatory cytokines, thereby enhancing phagocytosis, cytotoxic T-cell activity, and antitumor immunity. In contrast, exosomal cargo activates MAPK/STAT3 signaling and promotes M2 polarization, characterized by increased CD206, Arg-1, IL-10, TGF-β, MMP9, and VEGF expression. M2 macrophages further reinforce STAT3 signaling through an IL-6/IL-10-dependent feedback loop, ultimately promoting angiogenesis, metastasis, immune escape, and HCC progression.

Among the downstream mechanisms, STAT3 signaling serves as a central convergence point linking exosomal RNA cargo to M2 macrophage polarization and immunosuppressive cytokine production (72, 73). STAT3 activation drives the production of cytokines such as IL-6, IL-10, and VEGF. These cytokines not only induce M2 macrophage polarization but also act as feedback activators of STAT3 within the TME, thereby further amplifying immunosuppressive effects (74–76). Exosomes released by ER-stressed HCC cells can influence the cytokine expression profile of macrophages through the STAT3 signaling pathway. M2 macrophages secrete high levels of IL-6, IL-10, and monocyte chemoattractant protein-1 (MCP-1). This cytokine profile promotes macrophage-mediated immunosuppression, tumor-cell proliferation and invasion, and suppression of antitumor immunity (77, 78). *In vivo*, the negative regulatory effect of miR-146a on antitumor immune responses is mediated through STAT3 (79). After blockade of the STAT3 pathway, miR-146a expression was downregulated, levels of cytokines such as TGF-β, IL-6, and IL-18 decreased, whereas IFN-γ increased, resulting in improved immune function in mice. Conversely, increased miR-146 expression can upregulate inflammatory factors such as IL-6 and IL-8, as well as the immunosuppressive factor TGF-β, thereby activating STAT3 and generating a TME that promotes HCC progression. Therefore, targeting STAT3-mediated exosomal regulation of M2 macrophage polarization represents a promising strategy for inhibiting HCC progression (54, 80). These findings suggest several potential strategies, including inhibition of exosome release or uptake, blockade of oncogenic exosomal miRNAs or lncRNAs, suppression of STAT3-dependent signaling, and reprogramming of TAMs from an M2-like to an M1-like phenotype. Such approaches may enhance antitumor immunity and improve the efficacy of immune checkpoint blockade in HCC (**Figure 2**).

## 4 Conclusion

Exosome-mediated modulation of macrophages plays a central role in shaping the immunosuppressive landscape of the HCC TME. HCC-derived exosomes are enriched in miRNAs such as miR-21, miR-146a-5p, and miR-149-5p, as well as lncRNAs including LncMMPA and TUC339, which collectively reprogram macrophages toward an M2-like phenotype. These M2-like macrophages secrete immunosuppressive cytokines, growth factors, and angiogenic molecules, thereby promoting immune evasion, tumor proliferation, and metastatic dissemination. Conversely, induction of M1 macrophage polarization through exosomal cargo or pharmacological modulation may enhance antitumor immunity by activating cytotoxic T cells and promoting inflammatory cytokine production.

Overall, exosome-driven macrophage polarization represents a critical mechanism underlying HCC progression and immune escape. Therapeutically, targeting exosomal RNAs, or downstream pathways such as STAT3 and NF-κB may help reprogram TAMs, restore antitumor immunity, enhance the efficacy of immune checkpoint blockade, and suppress metastasis. Future research should further elucidate the effects of exosomal cargo on macrophage plasticity, validate molecular targets in clinically relevant preclinical models, and integrate exosome-based therapeutic approaches with existing immunotherapies. However, clinical translation remains challenging because exosomal cargo is highly heterogeneous, macrophage responses are context-dependent, and standardized methods for exosome isolation, target validation, delivery, and safety assessment are still required. Ultimately, a deeper understanding of exosome–macrophage crosstalk may transform the immunotherapeutic landscape of HCC by enabling the development of novel interventions that improve prognosis and survival.
